# Vanillic acid attenuates testosterone-induced benign prostatic hyperplasia in rats and inhibits proliferation of prostatic epithelial cells

**DOI:** 10.18632/oncotarget.19909

**Published:** 2017-08-03

**Authors:** Yunu Jung, Jinbong Park, Hye-Lin Kim, Dong-Hyun Youn, JongWook Kang, Seona Lim, Mi-Young Jeong, Gautam Sethi, Sung-Joo Park, Kwang Seok Ahn, Jae-Young Um

**Affiliations:** ^1^ Department of Science in Korean Medicine, Graduate School, Kyung Hee University, Dongdaemun-Gu, Seoul 02447, Republic of Korea; ^2^ Basic Research Laboratory for Comorbidity Regulation, College of Korean Medicine, Kyung Hee University, Dongdaemun-Gu, Seoul 02447, Republic of Korea; ^3^ Department of Pharmacology, Yong Loo Lin School of Medicine, National University of Singapore, Singapore 117600, Singapore; ^4^ Department of Herbology, College of Oriental Medicine, Wonkwang University, Iksan, Jeonbuk 54538, Republic of Korea

**Keywords:** benign prostatic hyperplasia (BPH), vanillic acid, 5α-reductase, android receptor, estrogen receptor α

## Abstract

Benign prostatic hyperplasia (BPH) is a common disease in the male population, especially in elderly men. Vanillic acid (VA), a dihydroxybenzoic derivative used as a flavoring agent, is reported to have an anti-inflammatory effect. However, there are no reports of its effects on BPH to date. BPH was induced with a pre-4-week treatment of daily subcutaneous injections of testosterone propionate (TP), and the normal control group received injections of ethanol with corn oil instead. Six weeks of further injections were done with (a) ethanol with corn oil, (b) TP only, (c) TP + finasteride, and (d) TP + VA. Finasteride was used as a positive control group. VA had protective effects on the TP-induced BPH. In the VA treatment group, the prostate weight was reduced, and the histological changes including the epithelial thickness and lumen area were restored like in the normal control group. Furthermore, in the VA treatment group, two proliferation related factors, high molecular weight cytokeratin 34βE12 and α smooth muscle actin, were significantly down-regulated compared to the TP-induced BPH group. The expressions of dihydrotestosterone and 5α-reductase, the most crucial factors in BPH development, were suppressed by VA treatment. Expressions of the androgen receptor, estrogen receptor α and steroid receptor coactivator 1 were also significantly inhibited by VA compared to the TP-induced BPH group. In addition, we established an *in vitro* model for BPH by treating a normal human prostatic epithelial cell line RWPE-1 with TP. VA successfully inhibited proliferation and BPH-related factors in a concentration-dependent manner in this newly established model. These results suggest a new and potential pharmaceutical therapy of VA in the treatment of BPH.

## INTRODUCTION

Benign prostatic hyperplasia (BPH) is one of the most common chronic diseases in the male population, in which the incidence increases gradually with age, and almost 50% of men over 50 suffer from BPH symptoms [1]. The absolute prevalence rates of BPH widely differ in studies based on the distinct nation, longitude, or population [2, 3]; however, according to a biopsy and cadaver study, it is clear that BPH is an age-related disease [4].

Yet, much remains unclear about the biology of BPH. It is known as a heterogenous disease, and the histologic variability of BPH patients makes personalized therapies a possibility [5]. Androgens are closely related to BPH because testicular androgens are essential in the development of BPH [6]. Among the androgens, dihydrotestosterone (DHT) seems to be the most crucial factor. According to several studies, the serum concentration of DHT is elevated in BPH patients than in unaffected men with similar ages [7]. In the prostate, 5α-reductase (5AR), the nuclear membrane-bound enzyme steroid, converts testosterone into DHT, the principal androgen of the prostate [6]. DHT is a more potent androgen than testosterone because of its higher affinity for the androgen receptor (AR) [8]. Inside prostate cells, testosterone and DHT both bind to the same receptor, AR, which results in increased transcription of androgen-dependent genes and ultimately stimulate protein synthesis [9]. This specific receptor, AR, is a type of nuclear receptor that is activated by the binding of androgens in the cytoplasm which then are translocated into the nucleus [10]. Expressions of androgen-regulated genes are affected by co-regulators including steroid receptor coactivator 1 (SRC-1), which modify the transcriptional activity of AR which could be related to BPH [11]. In addition to the 5AR-AR pathway, estrogen receptors (ERs) α and β are also known to regulate prostatic proliferation [12].

BPH is understood as a histological diagnosis. However, when associated with lower urinary tract symptoms, BPH becomes a clinical entity. Men with severely enlarged prostates suffer under obstructive and irritative symptoms including a decreased peak urinary flow rate, incomplete bladder emptying, and greater risks of acute urinary retention, all of which negatively impact the quality of life [13]. Incomplete voiding can result in the stasis of bacteria in the bladder residue and cause an increased risk of urinary tract infection [14]. Furthermore, acute or chronic urinary retention increases while the residual urinary volume expands and can eventually result in bladder hypotonia [15].

The most frequently prescribed medications for BPH are α-blockers and 5α-reductase inhibitors (5ARIs). Alpha-blockers decrease the blockage of the urine flow, therefore is chosen as an initial therapy in several countries [16]. However, they are not effective when it comes to the size of the prostate [17], and side effects such as orthostatic hypotension and headaches occur commonly [18]. As its name implies, 5ARI, the other treatment option, inhibits the function of 5AR. However, the effects of 5ARIs may take longer to appear than α-blockers [19], and even more, side effects including decreased libido and ejaculatory or erectile dysfunction have been reported [20]. Therefore, as the need for new therapeutic treatments is increasing, herbal remedies are commonly considered for the treatment of BPH [21]. The USA and some European countries have approved such medications including *Hypoxis rooperi* [22], *Prunus Africana* [23], and of course the most famous and dominant herbal therapy for BPH, *Serenoa repens* (Saw palmetto) [24, 25].

4-hydroxy-3-methoxybenzoic acid (vanillic acid, VA) is a dihydroxybenzoic derivative used as a flavoring agent. The highest amount of VA in plants is in the root of *Angelica sinensis* [26], which has been widely used in Traditional Korean Medicine for centuries especially for female health issues [27]. Currently, some studies have reported the positive effects of *Angelica sinensis* on prostate cancer [28, 29]. *Canrium schweinfurthii* Engl, the African olive, which also contains VA, was reported to have protective effects against prostate cancer [30]. In addition, methyl vanillate, an analogue of VA is reported to be a proliferation-inhibitor of several prostate cell lines, including prostate cancer cell lines LNCaP and DU145, a nontumorigenic fibroblast cell line GM-0637, a prostate epithelial carcinoma cell line TRAMP, and a benign prostatic hyperplasia cell line BPH-1 [31]. However, to date, no study on the effects of VA on BPH has been reported yet.

In this study, we show the effects of VA on BPH in testosterone propionate (TP)-induced BPH rats by measuring the prostate tissue weight, examining the histological changes and evaluating major factors involved in the pathogenesis of BPH. Then, we further confirm its effect at the cellular level by measuring cell proliferation and BPH-related factors in TP-induced proliferated RWPE-1 cells.

## RESULTS

### VA suppresses prostatic hyperplasia in TP-induced BPH rats

The prostate was dissected like in the abdominal incision picture (Supplementary Figure 1A). The ventral prostate (VP) and dorsolateral prostate (DLP) were dissected following the steps shown in Supplementary Figure 1B (ventral view) and C (dorsal view), respectively.

**Figure 1:**
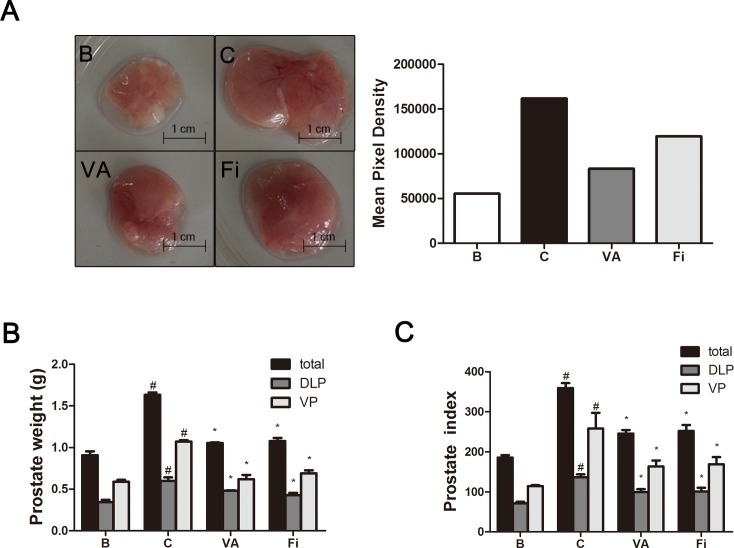
Effect of VA on prostate weight and prostate index in TP-induced BPH rats **(A)** Visual comparisons (upper panels) and area pixel density (lower panels) of the prostate tissues. **(B)** Total prostate, VP and DLP tissue weight, and **(C)** prostate indexes of rats. The prostate indexes were calculated dividing prostate weight (mg) by body weight (100 g). ^#^*P* < 0.05 when compared to B; ^*^P < 0.05 when compared to C. Total, total prostate; DLP, dorsolateral prostate; VP, ventral prostate; B, normal control group; C, TP-induced BPH group; VA, VA-treated BPH group; Fi, Fi-treated BPH group.

Figure 1A shows the size comparisons of the prostate tissues among the four groups. There was no significant difference in the body weights of the rats regardless of the TP treatment (data not shown). The TP-induced BPH group had a total prostate weight of 1756 ± 319 mg. This was significantly higher by 827 mg (1.88-fold change) when compared to the normal control group (938 ± 91 mg). The VA-treated group had a 568 mg decrease in the total prostate weight (1188 ± 185 mg), and the finasteride group (1232 ± 228 mg) had a decrease by 524 mg when compared to the TP group. The VP was bigger than the DLP in each group. The normal control, TP-induced BPH, VA-treated, and finasteride-treated groups had a VP weight of 578 ± 39 mg, 1196 ± 366 mg, 724 ± 156 mg, and 713 ± 68 mg and a DLP weight of 360 ± 52 mg, 600 ± 70 mg, 540 ± 21 mg, and 410 ± 54 mg, respectively (Figure 1C).

The prostate weight index was calculated dividing the body weight (100 g) by the prostate tissue (total, ventral, or dorsolateral) weight (mg). As shown in Figure 1D, administration of TP significantly elevated the total prostate weight index when compared with the normal control rats. Treatment with VA significantly decreased the total prostate weight index when compared to the TP-treated group. A similar effect was observed in the finasteride-treated group when compared to the TP-treated group. The percentage inhibition was found to be 31.55% and 29.78% by VA and finasteride, respectively, when compared with the TP-induced BPH group. Similar effects on the VP and DLP indexes were observed. Administration of TP also significantly elevated the VP and DLP indexes when compared with the normal control group. The VA and finasteride groups had significantly decreased VP and DLP indexes when compared to the TP-treated control group. The percentage inhibition was 39.33% and 34.73% on the VP index and 23.22% and 25.74% on the DLP index in the VA and finasteride group, respectively. As TP injection increased VP higher than DLP in both size and weight, our further investigation was performed using VP tissues.

### VA restores histological changes induced by TP-administration in the prostate tissues of TP-induced BPH rats

**Figure 2:**
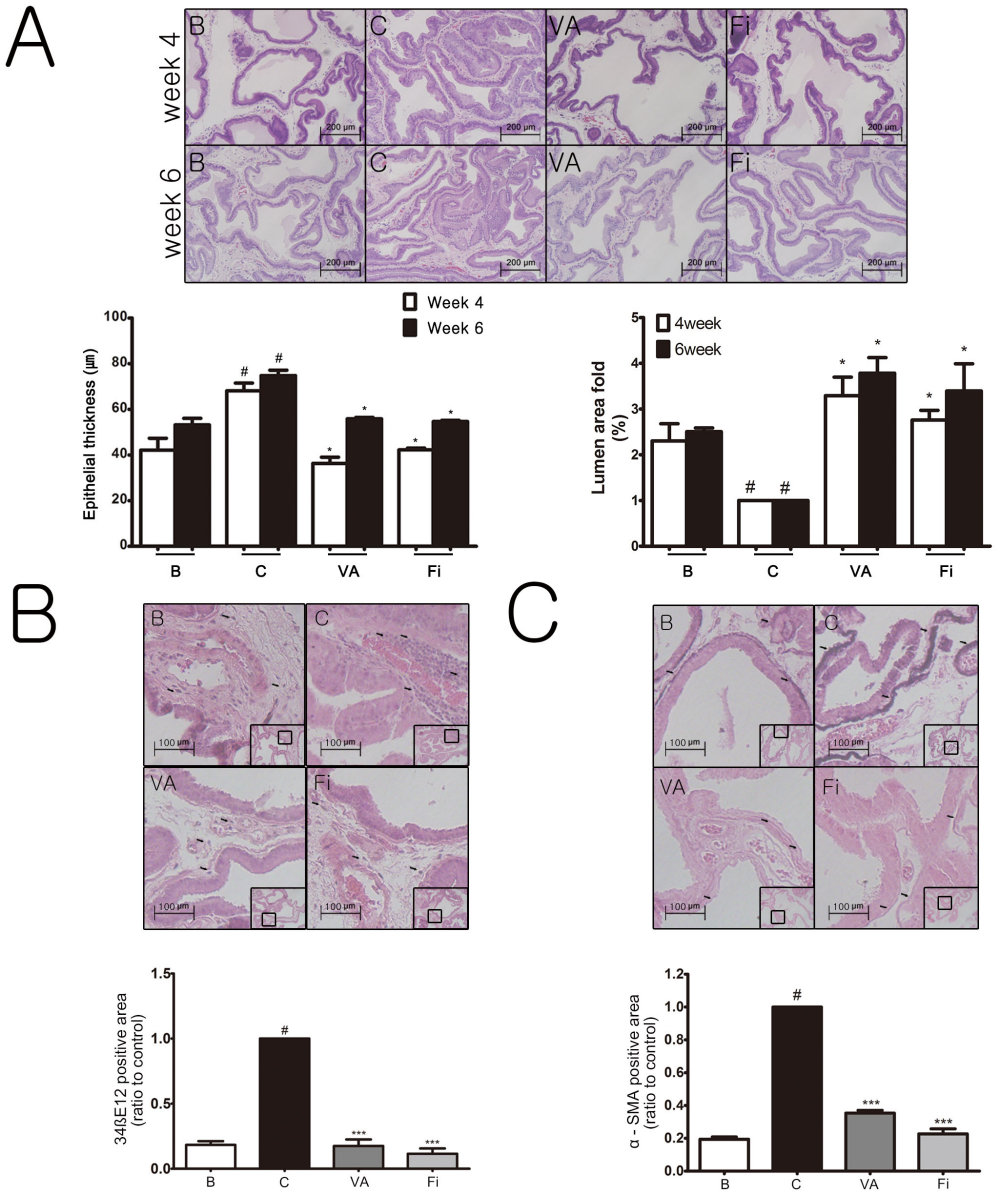
Effect of VA on histological changes of the prostate tissues in TP-induced BPH rats **(A)** Representative photomicrograph of H&E stained prostate tissues (magnification ×200), epithelial thickness and relative lumen area of the prostate tissues. Representative photomicrograph and relative density of IHC stained prostate tissues with antibodies against **(B)** 34βE12 and **(C)** αSMA (arrows indicate immunostained cells, magnification ×400). ^#^*P* < 0.05 when compared to B; ^*^P < 0.05 when compared to C; ^***^P < 0.001 when compared to C. B, normal control group; C, TP-induced BPH group; VA, VA-treated BPH group; Fi, Fi-treated BPH group.

We evaluated the changes in the histomorphology of the prostate tissues by hematoxylin & eosin (H&E) staining. Figure 2A shows the epithelial thickness of the prostate at different time points (week 4 and 6). The TP treatment group (68.10 ± 16.15 μm) produced a significant increase in the epithelial thickness of the prostates at week 4 by 26.00 μm compared to the normal rats (42.10 ± 12.33 μm). However, treatment with VA (36.30 ± 9.11 μm) significantly decreased the epithelial thickness at week 4 by 31.80 μm, respectively, when compared with the TP-treated group, while the finasteride group (42.23 ± 9.13 μm) reduced the thickness by 25.87 μm. The epithelial thickness at week 6 also showed similar results but with smaller gaps. The TP-treated group (74.77 ± 24.27 μm) significantly increased the epithelial thickness by 21.57 μm compared to the normal control group (53.20 ± 14.70 μm). On the other hand, the VA (55.83 ± 11.80 μm) and finasteride (54.67 ± 15.41 μm) groups had a significant decrease compared to the TP-administrated group by 18.94 μm and 20.10 μm, respectively. Administration of TP reduced the prostatic lumen area of the tissue cells when compared with the normal blank group. However, treatment with VA for 4 weeks or 6 weeks (Figure 2A) resulted in significant increases (*P* < 0.05) in the prostatic lumen areas when compared with the BPH group. Daily treatment of finasteride was also capable of increasing the prostatic lumen area compared to the TP-treated group. Because the results of week 6 showed greater hyperplasia than that of week 4, all further experiments were performed with the prostate samples from week 6.

### VA reduces the expressions of prostatic proliferation markers in the prostate tissues of TP-induced BPH rats

High molecular weight cytokeratin 34βE12 (34βE12) has previously been considered to be a good marker for prostatic carcinoma because it is expressed in the basal cells of the prostate epithelium but not in carcinoma because the basal cells disappear as prostatic carcinoma proceeds. According to Mahopokai *et al*. [32], 34βE12 is positively indicated in BPH tissues by immunostaining, especially around the area of inflammation. The elevated level of 34βE12 may imply the proliferation of non-carcinoma cells. As seen in Figure 2B, 34βE12 was elevated in the TP-treated group when compared with the normal group. The VA and finasteride treatment suppressed the elevation of 34βE12 down to nearly the normal control group. Although the etiology of BPH is not fully understood, the morphometrical predominance of fibromuscular stroma suggests that it is primarily caused by an unproportional hyperproliferation of prostate stromal cells [33, 34]. Among the stromal cell subtypes, activated α-smooth muscle actin (αSMA)-positive myofibroblasts are the major source of connective tissues in BPH [35, 36]. Consistent to previously reported studies, the expression of αSMA was upregulated up to a 5-fold change by the TP treatment, creating a band-like form around the epithelial but was reduced by both the VA and finasteride treatment (Figure 2C).

### VA suppresses serum DHT and prostatic 5AR-2 in TP-induced BPH rats

**Figure 3:**
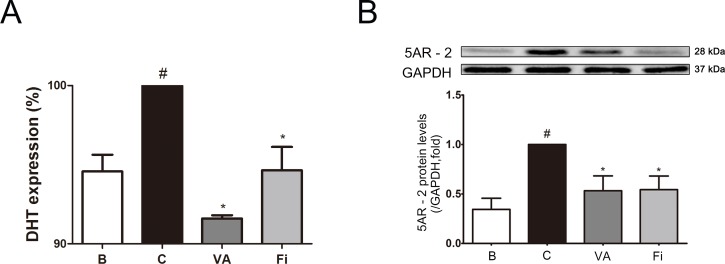
Effect of VA on serum DHT and prostatic 5AR-2 in TP-induced BPH rats **(A)** Serum ELISA for DHT. **(B)** Representative western blot bands and normalized relative PSA expression of each group. The differences in protein expressions were normalized to GAPDH. Values are the mean ± S.D. of the data from three or more separate experiments. ^#^*P* < 0.05 when compared to B; ^*^P < 0.05 when compared to C; ^**^P < 0.01 when compared to C. B, normal control group; C, TP-induced BPH group; VA, VA-treated BPH group; Fi, Fi-treated BPH group.

Next, we evaluated the DHT levels, one of the most crucial factors in BPH development in the serum of the rats. By TP administration, the serum DHT level showed a 5.74% increase when compared to the normal control rats. The increased DHT level was reduced by 8.40% in the VA-treated rats, which was higher than the 5.35%-reduced finasteride-treated rats (Figure 3A).

To examine the 5AR-2 differences among the groups, a western blot analysis was performed. As shown in Figure 3B, the expression of 5AR-2 was elevated up to nearly 3 times higher by the TP treatment compared to the normal control group. On the other hand, treatment with VA showed an inhibition rate of 46.73% even though the rats received the same TP treatment. This inhibition rate of VA was even higher than that of the finasteride treatment group (45.64%).

### VA reduces the expression of AR, ER and SRC1 in the prostate tissues of TP-induced BPH rats

**Figure 4:**
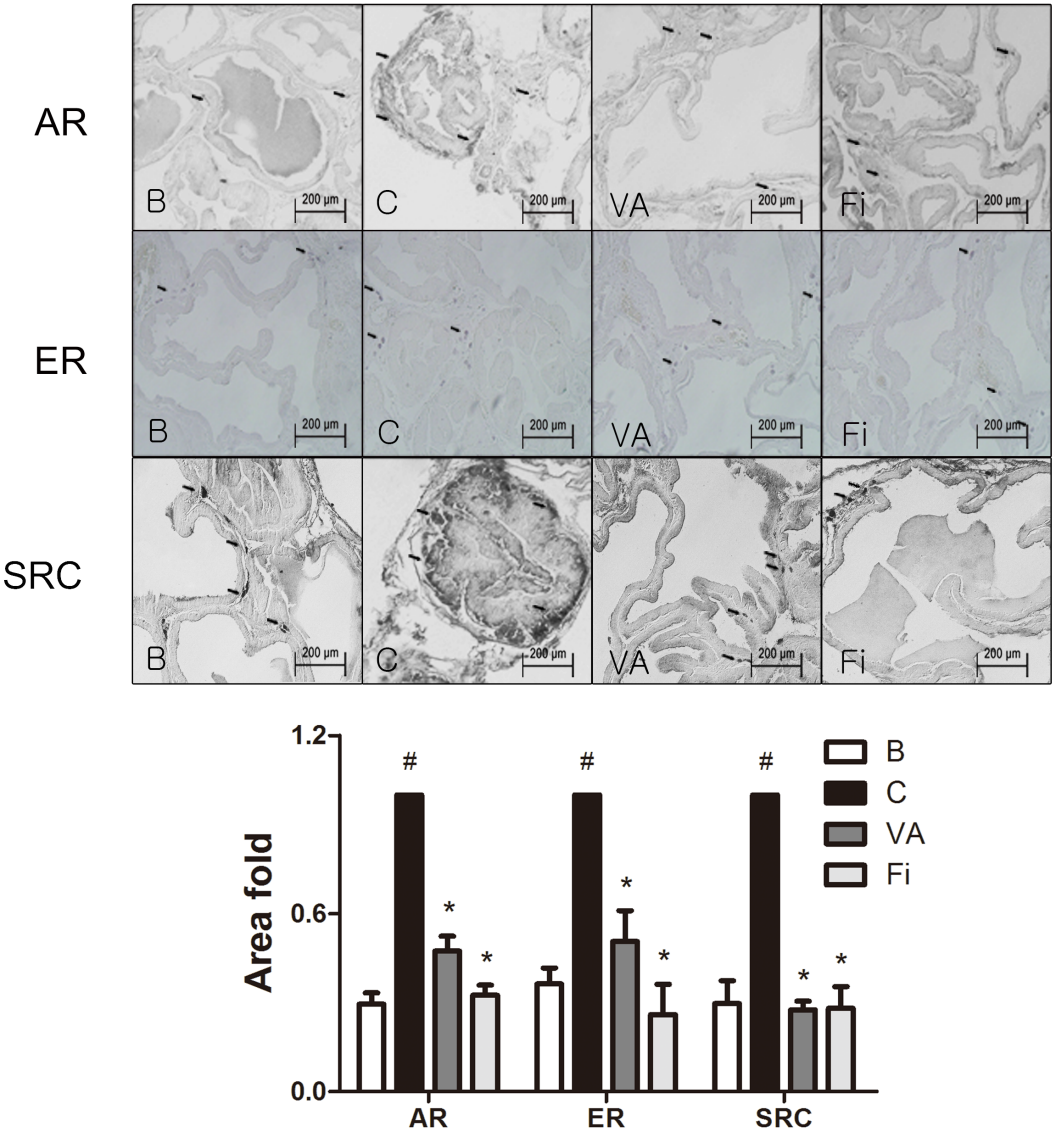
Immunohistochemical analysis of AR, ERα and SRC1 in the prostate tissues of TP-induced BPH rats Representative photomicrographs of the immunohistochemically stained prostate tissues (upper panels, magnification ×400) and relative density of the positively immunostained area (lower panels) of AR, ERα and SRC1 of each group. Values are the mean ± S.D. of the data from three or more separate experiments. ^#^*P* < 0.05 when compared to B; ^*^P < 0.05 when compared to C. B, normal control group; C, TP-induced BPH group; VA, VA-treated BPH group; Fi, Fi-treated BPH group.

To evaluate effect of VA on BPH-related factors, immunohistochemistry (IHC) staining was performed in the prostate tissues of TP-induced BPH rats. AR, one of the major key factors in the development of BPH, was evaluated by microscopic examination of immunostained prostate slides. The TP-treated group showed elevated expression levels of AR when compared with the normal control group (Figure 4, upper panels). VA and finasteride treatment both suppressed the expression of AR at a rate of 52.66% and 67.58%, respectively. Substantial evidence has shown that stimulation of ERα and ERβ in the prostate can drive either proliferation or anti-proliferative mechanisms, respectively [12]. The immunostained prostate tissues showed an elevated level of ERα in the TP-induced BPH group. However, with the VA treatment, the ERα expression was decreased by 49.40% compared to the TP-treated group, while the finasteride-treated group showed a decreased expression of ERα by 74.05% (Figure 4, lower panels). SRC1 is the first identified member of the steroid receptor action regulators [37]. It interacts with AR and enhances both ligand-dependent and independent transactivation to increase the transcription of androgen-regulated genes [38]. As seen in Figure 4, the SRC1 positive area was highly upregulated by the TP treatment when compared to the normal control group. VA treatment decreased the SRC1 expression by a rate of 72.50%, which was comparable to the decrease rate of 71.97% by the finasteride treatment.

**Figure 5:**
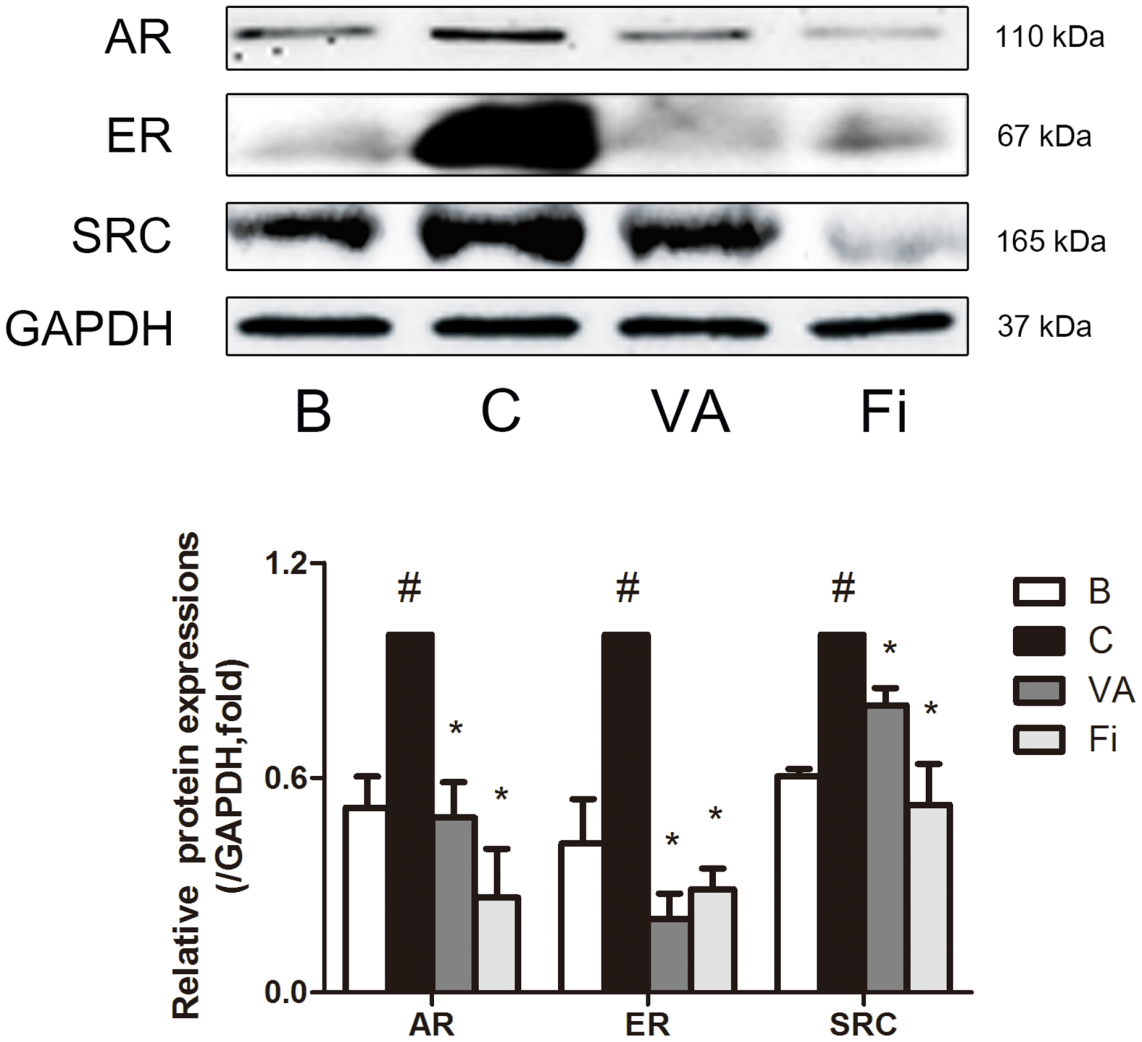
Effect of VA on the protein expressions of AR, ERα and SRC1 in the prostate tissues of TP-induced BPH rats Representative western blot bands (upper panels) and normalized relative expressions (lower panels) of AR, ERα and SRC1 of each group. The differences in protein expressions were normalized to GAPDH. Values are the mean ± S.D. of the data from three or more separate experiments. ^#^*P* < 0.05 when compared to B; ^*^P < 0.05 when compared to C. B, normal control group; C, TP-induced BPH group; VA, VA-treated BPH group; Fi, Fi-treated BPH group.

Then, to confirm the IHC results, a western blot analysis was performed. As shown in Figure 5, both the VA-treated group and finasteride-treated group had a decreased protein expression of AR with an inhibition rate of 51.13% and 73.44%, respectively, while the AR protein expression in the TP-induced BPH group was doubled compared to the normal control group. The protein level of ERα was also measured by western blot. With TP administration, ERα had a 2.4-fold increase, which was inhibited by 79.56% and 71.19% in the VA-treated group and finasteride-treated group, respectively. An additional western blot was done to evaluate the protein expression of SRC1. Consistent with the IHC results, SRC1 was elevated by the TP treatment, and the VA and finasteride treatment reduced the SRC1 expression by 38.63 % and 47.54%, respectively.

### VA suppresses cell proliferation and BPH-related factors in TP-induced proliferated RWPE-1 cells

**Figure 6:**
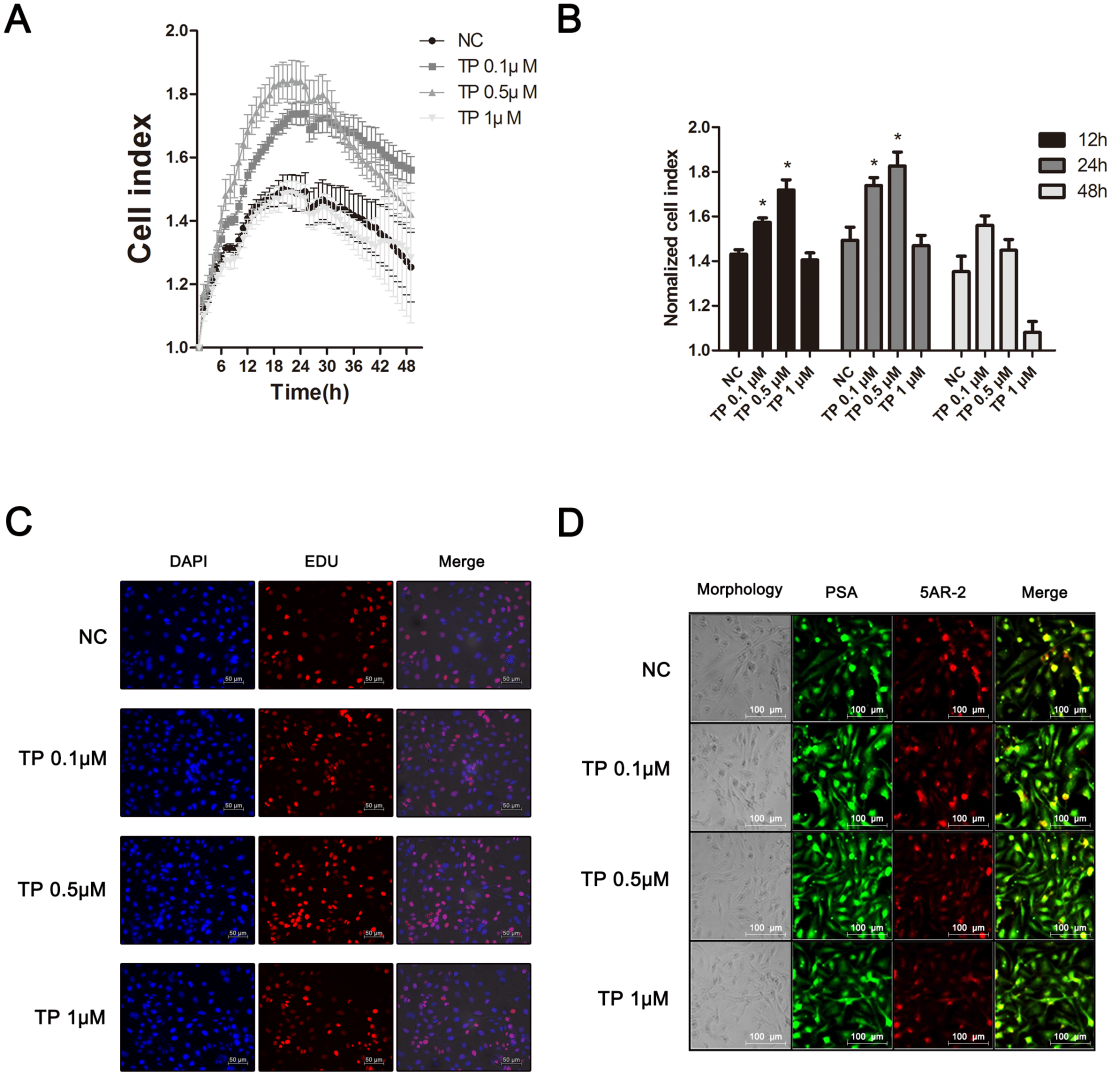
Effect of TP on proliferation and BPH-related factors in normal human prostatic epithelial RWPE-1 cells **(A)** Effect of various concentrations of TP on cell proliferation in RWPE-1 cells. **(B)** Normalized cell index at the time points 12, 24 and 48 h. Representative photomicrographs of the **(C)** EdU assay and **(D)** IF staining of PSA and 5AR-2 (magnification ×200). Values are the mean ± S.D. of the data from three or more separate experiments. ^*^P < 0.05 when compared to NC. NC, non-treated RWPE-1 cells.

Next, we established a *vitro* model for BPH using normal prostate epithelial RWPE-1 cells for further studies. Results from our previous report showed that TP treatment induced the proliferation of RWPE-1 cells [39]. To find the most effective concentration of TP, we treated the RWPE-1 cells with various concentrations of TP and evaluated the changes. Figure 6A and 6B shows the real time cell analyzer (RTCA) results in which the proliferation of RWPE-1 cells was induced by 0.1 and 0.5 μM of TP. However, at 24 h after the initial TP treatment, the TP-treated and non-treated RWPE-1 cells showed a time-dependent decline in cell proliferation. Additionally, 1 μM of TP-administration did not affect RWPE-1 cell proliferation. An EdU assay was performed to confirm the effect of TP. Similar to the RTCA results, the TP treatment at 0.1 and 0.5 μM boosted the proliferation of cells, which was higher by 0.5 μM of TP (Figure 6C). In addition, we observed up-regulated prostatic specific antigen (PSA) and 5AR-2 levels induced by the 0.1 and 0.5 μM TP treatment in the RWPE-1 cells (Figure 6D). PSA, a glycoprotein enzyme, is a widely-used marker for the diagnosis of BPH [40]. Through these results, we did further assays using the BPH-like *vitro* model established by a 0.5 μM TP treatment for 24 h in RWPE-1 cells.

**Figure 7:**
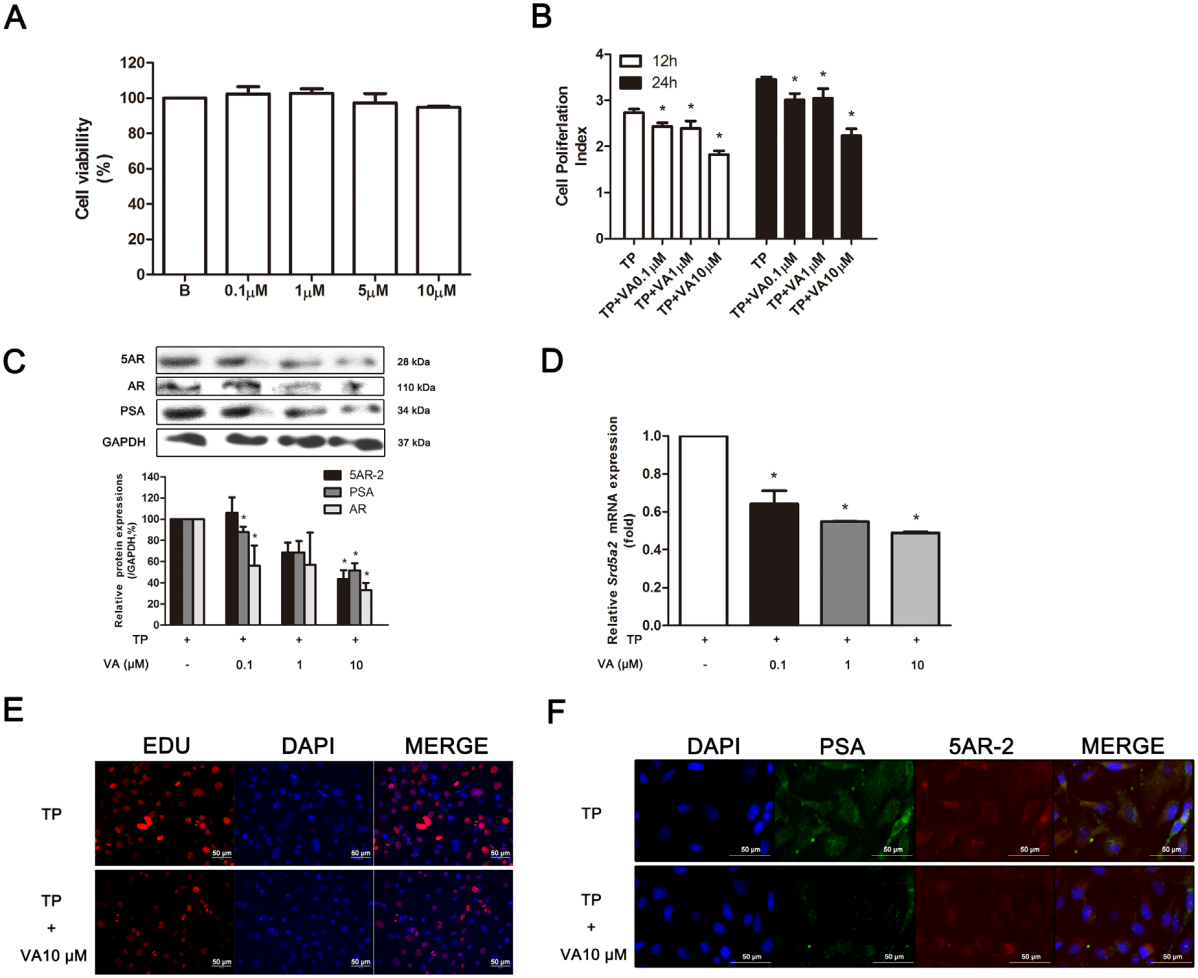
Effect of VA on TP-induced proliferated RWPE-1 cells **(A)** Effect of VA on the cell viability of RWPE-1 cells. **(B)** Effect of VA on cell proliferation in TP-induced proliferated RWPE-1 cells at the time points of 12 and 24 h. **(D)** Representative western blot bands (upper panels) and normalized relative expressions (lower panels) of AR, ERα and SRC1 of each group. **(E)** Effect of VA on *Sdr5ar2* mRNA level in TP-induced proliferated RWPE-1 cells. Representative photomicrographs of the **(F)** EdU assay and **(C)** IF staining of PSA and 5AR-2 (magnification ×200). The differences in protein expressions were normalized to GAPDH. Values are the mean ± S.D. of data from three or more separate experiments. ^*^P < 0.05 when compared to TP. TP, 0.5 μM TP-treated RWPE-1 cells.

Before evaluating the effects of VA on the RWPE-1 cells, we first assessed whether it affects the viability of the RWPE-1 cells. As seen in Figure 7A, up to 10 μM of VA did not show any cytotoxicity in the RWPE-1 cells. Therefore, further assays were performed with 0.1, 1 and 10 μM of VA. The VA also in a concentration dependent manner inhibited the cell proliferation induced by the TP, which was observed in the RTCA results (Figure 7B). Western blot showed that VA reduced the expressions of PSA, AR and 5AR-2 in the TP-treated RWPE-1 cells in a concentration dependent manner (Figure 7C). The suppressive effect of VA was affective on the mRNA level. A real-time RT-PCR assay showed that VA treatment concentration-dependently reduced *Srd5a2*, the gene which encodes 5AR (Figure 7D). By an EdU assay, we could confirm VA treatment significantly inhibited TP-induced proliferation of RWPE-1 cells (Figure 7E). In addition, immunofluorescence (IF) staining confirmed the western blot results because PSA, a factor for the diagnosis of prostatic proliferation, and 5AR-2, the key enzyme in BPH pathogenesis, were both highly reduced by 10 μM of VA treatment in the TP-induced proliferated RWPE-1 cells (Figure 7F).

## DISCUSSION

Currently there are no completely effective treatments for BPH [41]. The two major classes of drugs mainly prescribed to treat BPH are α-blockers and 5ARIs. Alpha-blockers, including doxazosin, terazosin, tamsulosin and alfuzosin, relax the smooth muscle fibers of the prostate and thereby, reduce the dynamic components of prostatic obstruction [42]. 5ARIs, such as finasteride and dutasteride, decrease the levels of intracellular DHT without reducing the testosterone levels leading to a 20-30% reduction in the prostate size [43]. However, the number of patients who consider alternative medication is increasing [25] because on a whole, phytotherapeutic drugs have remarkably benign adverse effects [44]. Besides the most widely used BPH alternative medication Saw palmetto, several prescriptions or herbs from Traditional Korean Medicine have been reported to be effective for BPH, such as Yukmijihwang-tang [45], *Rubus coreanus* [46], *Scutellaria baicalensis* [47], *Curcuma longa* [48], *Phellodendron amurense* [49] and *Cinnamomi cortex* [50].

While VA is a compound derived from *Angelica sinensis*, olives [51], rice [52], ginsengs [53], acai fruits [54], and various kinds of berries are also rich in VA [55]. Despite the various reports on the properties of VA, there has not been one study on the effects of VA on BPH. In this study, we investigated the effect of VA on BPH using TP-induced BPH rats and proliferated RWPE-1 cells. Since Kato *et al*. have first reported increased prostate weight by treatment with TP [56], the rat model of BPH has been developed and widely used in BPH studies. Based on the detailed human studies of McNeal [57, 58], several homologies offer an opportunity to examine animal BPH models with the premise of understanding the mechanisms and etiology of the pathological processes involved in BPH. In addition, Lesovaya et al. described two different rat models for BPH study: a TP-induced BPH model of which enlargement usually occurs in VP region, and a sulpiride-induced BPH model, a model which hyperplasia usually appears in the DLP region of the prostate [59].

5AR enzymes, also known as 3-oxo-5α-steroid 4-dehydrogenases, are enzymes involved in steroid metabolism. Two steroid 5AR enzymes have been discovered and are encoded by separate genes; the type I 5α reductase (5AR-1) is predominant in extra-prostatic tissues while type II 5α reductase (5AR-2) is predominant in prostate tissues [60]. The androgen axis is targeted by the inhibitors of 5ARs to prevent the conversion of testosterone to DHT [61]. Inhibition of 5AR results in decreased conversion of testosterone to DHT leading to the inhibition of prostatic proliferation. Unlike other androgen-dependent organs, the prostate maintains its androgen-responding ability throughout life, and the level of AR remains high while aging [62]. In the present study, VA had a significant suppressive effect on 5AR-2. The decrease rate was even higher when compared to finasteride treatment. In addition, VA also down-regulated the level of AR and its co-regulator SRC1. Because 5AR is the initial trigger of prostatic hyperplasia and AR is the main receptor in that process, these results suggest the pharmaceutical potential of VA as a therapeutic agent for BPH.

In addition to the down-regulation of 5AR and AR, VA administration also suppressed the level of ERα compared to that of the BPH group, which may suggest another potential action mechanism of VA on BPH treatment besides the 5AR-AR axis. Whereas the role of androgens in BPH has been studied extensively for decades, the effect of estrogens has only gained interest recently [63]. An increased expression of ERα with a concomitant decrease in ERβ has been shown to be correlated with BPH and other prostate-proliferating diseases, respectively [12, 64]. In the aging male, there is a continued decrease in the ratio of circulating levels of androgens to estrogens [65]. VA successfully reduced ERα expression when assessed by western blot and IHC staining suggesting a possibility that the BPH-improving effect of VA may also be due to a mechanism involving ER action.

Despite the various animal experiment models of prostatic hyperplasia widely used for BPH studies, only few *in vitro* models have been introduced. We established a cell model for BPH studies using normal prostate epithelial RWPE-1 cells from our previous studies [39, 50]. In this study, we provide evidence for the suitability of this model for BPH study by showing TP-induced proliferation and increases in BPH-related factors in RWPE-1 cells. We also showed that VA decreased the proliferation of RWPE-1 cells by regulating BPH-related factors including 5AR-2, AR and ERα suggesting its BPH-inhibiting action is cell autonomous. Results from our newly established experimental model provide a possibility that the TP-induced RWPE-1 cells may be appropriate for *in vitro*-based BPH studies. The induced proliferation and expressions of PSA, 5AR-2 and AR clearly indicate the TP treatment in RWPE-1 cells causes cellular changes which resemble the pathogenesis of BPH development. Our next interest will be focused on the 5AR-AR axis of this particular cell model in an attempt to reveal the molecular mechanism underlying the pathogenesis of BPH.

In this study, we have shown that VA has suppressive effects on TP-induced prostatic enlargement in rats. The weight of the prostate tissues was increased by TP treatment while VA treatment reduced it. The histological changes such as epithelial thickness and lumen area by TP treatment were also restored by VA like those of the normal prostate group. 34βE12 and αSMA, prostate proliferation factors, were upregulated by TP injection, which were also reduced by VA treatment. In addition, TP treatment induced the elevation of the central enzyme of BPH, 5AR; however, VA treatment down-regulated the expression rate. The main BPH-related receptors, AR and ERα, and the AR-coactivating protein SRC1 were also upregulated in the TP group and inhibited by the VA treatment. The positive effects of VA on prostate hyperplasia were demonstrated in this study. These results indicate a new potential for pharmacotherapy treatment of BPH.

## MATERIALS AND METHODS

### Chemical reagents

VA (≥ 97% pure) was purchased from Sigma-Aldrich Inc. (St. Louis, MO, USA) and dissolved in 100 % dimethyl sulfoxide (DMSO). TP was provided by Wako pure chemical industries (Osaka, Japan), and finasteride (≥ 97% pure) was purchased from Sigma-Aldrich Inc. Antibodies for AR, ERα and SRC1 were purchased from Pierce biotechnology (Rockford, IL, USA); antibodies for 5AR-2 and 34βE12 were purchased from Abcam Inc. (Cambridge, MA, USA); the antibody for ARA70 was purchased from Aviva systems biology (San Diego, CA, USA), and the antibody for αSMA was purchased from Sigma-Aldrich Inc.

### Animals

12-week-old male Sprague-Dawley (SD) rats (*n* = 32) with initial body weights of 200-220 g were purchased from the Dae-Han Experimental Animal Center (Dae-Han Biolink, Eumsung, Korea). All animal experiments were performed in accordance with the ethical regulations of Kyung Hee University and approved by the Institutional Review Board of Kyung Hee University (confirmation number: KHUASP(SE)-P-034). The rats were housed in a pathogen-free room maintained at 23 ± 2°C with a relative humidity of 70% and an alternating 12 h light/dark cycle. Water and a standard laboratory diet (CJ Feed Co., Ltd., Seoul, Korea) were provided *ad libitum*.

BPH was induced by a pre-4-week treatment of daily subcutaneous injections of TP (5 mg/kg) at the inguinal region (*n* = 24). To establish the control group, 8 rats received ethanol with corn oil instead of TP. After the pre-treatment of 4 weeks, the BPH induced rats were randomly divided into three groups, and the rats that did not receive TP treatment became the normal control group. Thus, the rats were divided into four groups: (a) a normal control group that received ethanol with corn oil; (b) a BPH group that received TP with corn oil; (c) a positive control group that received finasteride (1 mg/kg), which is a 5ARI frequently used as a treatment for BPH [66], with TP (5 mg/kg); and (d) a group that received VA (5 mg/kg) with TP (5 mg/kg). To compare the effect of VA with different administration times, the rats were sacrificed at two time points; at week 4 and 6. VA and Fi were administered to animals once daily for 4 or 6 weeks following the pre-4-week BPH inducement. After the final treatment, the animals were fasted overnight and euthanized using CO_2_. Blood samples were obtained from the caudal vena cava. The blood containing tubes remained at RT for 2 h, and the sera were separated by centrifuging at 3000 ×*g* for 20 min at 4°C. The serum was stored at −80°C for further assays. The intact prostate tissues were carefully dissociated and removed, washed with PBS, and then weighed. The relative prostate weight (prostate index) was calculated as the ratio of prostate weight (g) to body weight (100 g). The prostate tissues were divided in half; one half was fixed in 10% formalin and embedded in paraffin for histomorphological assays; the other half was stored at −80°C for further assays.

### H&E and IHC staining

The formalin-fixed, paraffin-embedded prostate specimens were cut into 4-μm-thick tissue sections and prepared for further staining as previously described [50, 67]. For H&E staining, the sections were stained in hematoxylin for 5 min and then washed with water for 5 min. Then, the sections were stained in eosin for 30 s, dehydrated and mounted by routine methods, and the histological changes were observed with an Olympus IX71 Research Inverted Phase microscope (Olympus Co., Tokyo, Japan). For IHC staining, sections were incubated at 4°C overnight with a 1:50 dilution of the primary antibody for AR, ERα, SRC1, 5AR, αSMA or 34βE12 and then incubated at room temperature for 30 min with a 1:500 dilution of the horseradish peroxidase (HRP)-conjugated Affinipure Goat anti-rabbit IgG (Jackson Immunoresearch lab., PA, USA) or HRP-conjugated Affinipure Goat anti-mouse IgG (Jackson Immunoresearch lab., PA, USA). Following the addition of the detection system, the reaction was visualized with diaminobenzidine (DAB) in the presence of hydrogen peroxide. The slides were examined with the Olympus IX71 Research Inverted Phase microscope (Olympus Co.), and the density was measured with the ImageJ 1.47v software (National Institute of Health, Bethesda, MD, USA).

### DHT assay

Serum DHT levels of the rats were measured with a rat DHT ELISA kit according to the manufacturer's instructions (SunLong Biotech Co., Hangzhou, China). Briefly, standards and plasma samples from the rats were added to the DHT-antibody-pre-coated MicroElisa stripplates, and then, HRP-conjugated antibodies were added. The OD value was measured at 450 nm with a VERSAmax microplate reader (Molecular Devices LLC, Sunnyvale, CA, USA).

### Real time RT-PCR

Real-time RT-PCR analyses were performed described previously [68]. Briefly, total RNA from RWPE-1 cells were isolated using a QIAzol lysis reagent (QIAGEN sciences Inc., Venlo, Netherlands) and a GeneAllR RiboEx Total RNA extraction kit (GeneAll Biotechnology, Seoul, Korea). Total RNA was used as a template for first strand cDNA synthesis by a Power cDNA synthesis kit (iNtRON Biotechnology, Seoul, Korea). Step One Plus Real-Time PCR System (Applied Biosystems, Foster City, CA, USA) was used for PCR analysis. The primers for *Srd5ar2* were Forward 5′ TCC CGC TTG GCC TTT TG-3′ and Reverse 5′- GCC GTT ACC CTC CTT GTT TTC-3′.

### Western blotting

Western blotting was performed as previously described [39, 69]. Prepared prostate tissues were cut into pieces and homogenized with the Bullet Blender homogenization kit (Next Advance Inc., Averill Park, NY, USA). Homogenized tissues or cells were lysed with ice-cold RIPA buffer. The proteins in the supernatants were separated by sodium dodecyl sulfate-polyacrylamide gel electrophoresis and transferred onto polyvinylidenedifluoride (PVDF) membranes. The membranes were washed with TBST and then incubated with the appropriate primary antibodies at 4°C overnight. The blots were subsequently incubated with HRP-conjugated Affinipure Goat anti-rabbit IgG (Jackson Immunoresearch lab., West Grove, PA, USA) or HRP-conjugated Affinipure Goat anti-mouse IgG (Jackson Immunoresearch lab). The chemiluminescent intensities of the protein signals were quantified with the ImageJ 1.47v software (National Institute of Health).

### Cell culture

The normal human prostatic epithelial cell line RWPE-1 cells (American Type Culture Collection, Manassas, VA, USA) were cultured as previously described [39]. Briefly, RWPE-1 cells were cultured for 24 h in Roswell Park Memorial Institute medium (RPMI) (Gibco, Big Cabin, OK, USA) supplemented with 100 mg/ml penicillin/streptomycin (HyClone, Logan, UT, USA) and 10% FBS (Sigma-Aldrich Inc.). Then, the culture medium was replaced with fresh medium containing 0.5 μM of TP to induce cell proliferation. VA was supplemented together within the TP-containing medium.

### EdU proliferation assay

An EdU assay was performed with the Click-iT EdU Imaging Kit (Invitrogen, Waltham, MA, USA) as previously described [39]. Samples were analyzed with the iRiS Digital Cell Imaging System (Logos Biosystems, Anyang, Korea).

### IF staining

For PSA and 5AR-2 immunofluorescence evaluation, VA-treated cells were washed with PBS, fixed in 4% formaldehyde and permeabilized with PBS containing 0.25% Triton X-100 for 10 min. After that, non-specific binding was blocked with 5% BSA in PBS, and then, the cells were incubated with UCP1 and PGC1α antibody in 5% BSA in PBS overnight at 4°C. After that, they were incubated with the fluorescent secondary antibody Alexa Fluor 488 (Thermo Fisher Scientific, Waltham, MA, USA) and Alexa Fluor 546 (Thermo Fisher Scientific) followed by incubation with DAPI for nuclei staining. Images were acquired with the iRiS Digital Cell Imaging System (Logos Biosystems, Anyang, Korea).

### MTS assay

Cell viability of the RWPE-1 cells were assayed with the cell proliferation MTS kit (Promega, Madison, WI, USA) as described previously [39]. The OD value was measured at 500 nm with a VERSAmax microplate reader (Molecular Devices LLC).

### RTCA assay

Cell proliferation of the RWPE-1 cells was monitored with an xCELLigence RTCA MP Instrument (Roche Diagnostics GmbH, Berlin, Germany) for 48 h. Background impedance was measured in 100 μL of culture medium, and the final volume was adjusted to 200 μL (5 × 10^3^ cell/well). The impedance was recorded every 15 min. The cell index was normalized to the impedance at the time point of the TP or TP + VA administration.

### Statistical analysis

The data values are presented as the mean ± S.D. Differences in mean values were analyzed by one-way ANOVA or one-tailed Student's *t* test with the IBM SPSS Statistics 22 software (International Business Machines Corp., Armonk, NY, USA). Values with a *P* < 0.05 were considered to indicate statistical significance.

## SUPPLEMENTARY MATERIALS FIGURE



## Abbreviations

34βE12: high molecular weight cytokeratin 34βE12
5AR: 5α-reductase
5ARI: 5α-reductase inhibitor
αSMA: α-smooth muscle actin
AR: androgen receptor
BPH: benign prostatic hyperplasia
DHT: dihydrotestosterone
DLP: dorsolateral prostate
ER: estrogen receptor
SRC1: steroid receptor coactivator 1
TP: testosterone propionate
VA: vanillic acid
VP: ventral prostate
