# Correction: Vanillic acid attenuates testosterone-induced benign prostatic hyperplasia in rats and inhibits proliferation of prostatic epithelial cells

**DOI:** 10.18632/oncotarget.27105

**Published:** 2019-07-16

**Authors:** Yunu Jung, Jinbong Park, Hye-Lin Kim, Dong-Hyun Youn, JongWook Kang, Seona Lim, Mi-Young Jeong, Gautam Sethi, Sung-Joo Park, Kwang Seok Ahn, Jae-Young Um

**Affiliations:** ^1^ Department of Science in Korean Medicine, Graduate School, Kyung Hee University, Dongdaemun-Gu, Seoul 02447, Republic of Korea; ^2^ Basic Research Laboratory for Comorbidity Regulation, College of Korean Medicine, Kyung Hee University, Dongdaemun-Gu, Seoul 02447, Republic of Korea; ^3^ Department of Pharmacology, Yong Loo Lin School of Medicine, National University of Singapore, Singapore 117600, Singapore; ^4^ Department of Herbology, College of Oriental Medicine, Wonkwang University, Iksan, Jeonbuk 54538, Republic of Korea

**This article has been corrected:** Due to errors in image processing, an IHC staining slide of ER in Figure 4 was used twice by mistake. The proper Figure 4 is shown below. The authors declare that these corrections do not change the results or conclusions of this paper.

 Original article: Oncotarget. 2017; 8:87194–87208. 87194-87208 . https://doi.org/10.18632/oncotarget.19909

**Figure 4:**
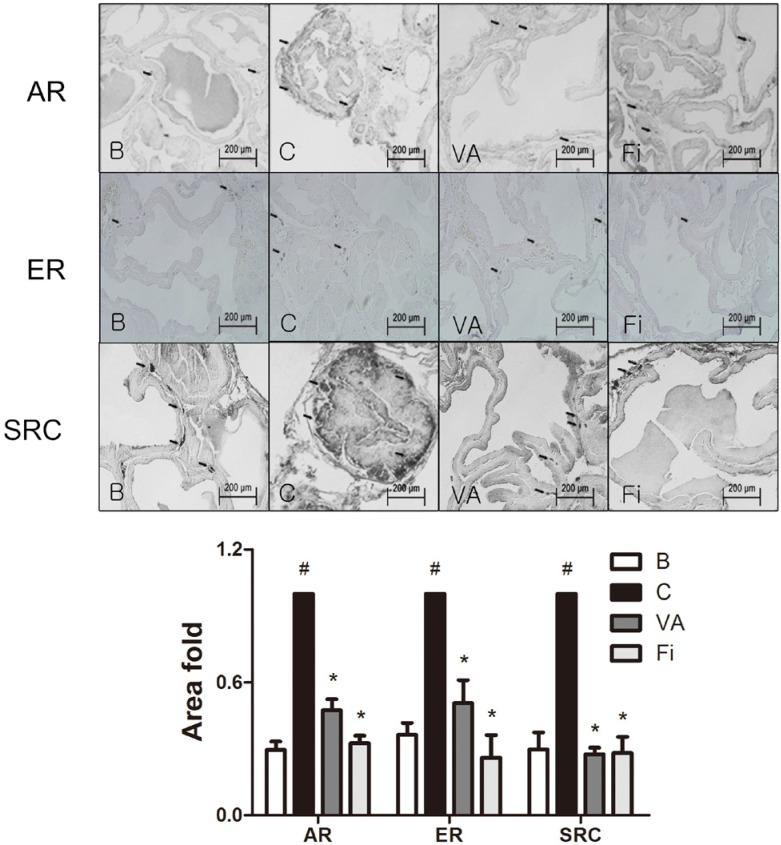
Immunohistochemical analysis of AR, ERα and SRC1 in the prostate tissues of TP-induced BPH rats. Representative photomicrographs of the immunohistochemically stained prostate tissues (upper panels, magnification ×400) and relative density of the positively immunostained area (lower panels) of AR, ERα and SRC1 of each group. Values are the mean ± S.D. of the data from three or more separate experiments. ^#^ *P* < 0.05 when compared to B; *P < 0.05 when compared to C. B, normal control group; C, TP-induced BPH group; VA, VA-treated BPH group; Fi, Fi-treated BPH group.

