# Supplementary material for: Vanillic acid attenuates testosterone-induced benign prostatic hyperplasia in rats and inhibits proliferation of prostatic epithelial cells
Source: Oncotarget. 2017 Aug 3;8(50):87194–208. doi: 10.18632/oncotarget.19909 (PMC5675626; doi:10.18632/oncotarget.19909)
Supplement: Supplementary file 1 [file oncotarget-08-87194-s001.pdf]

Vanillic acid attenuates testosterone-induced benign prostatic hyperplasia in rats and inhibits proliferation of prostatic epithelial cells

SUPPLEMENTARY MATERIALS

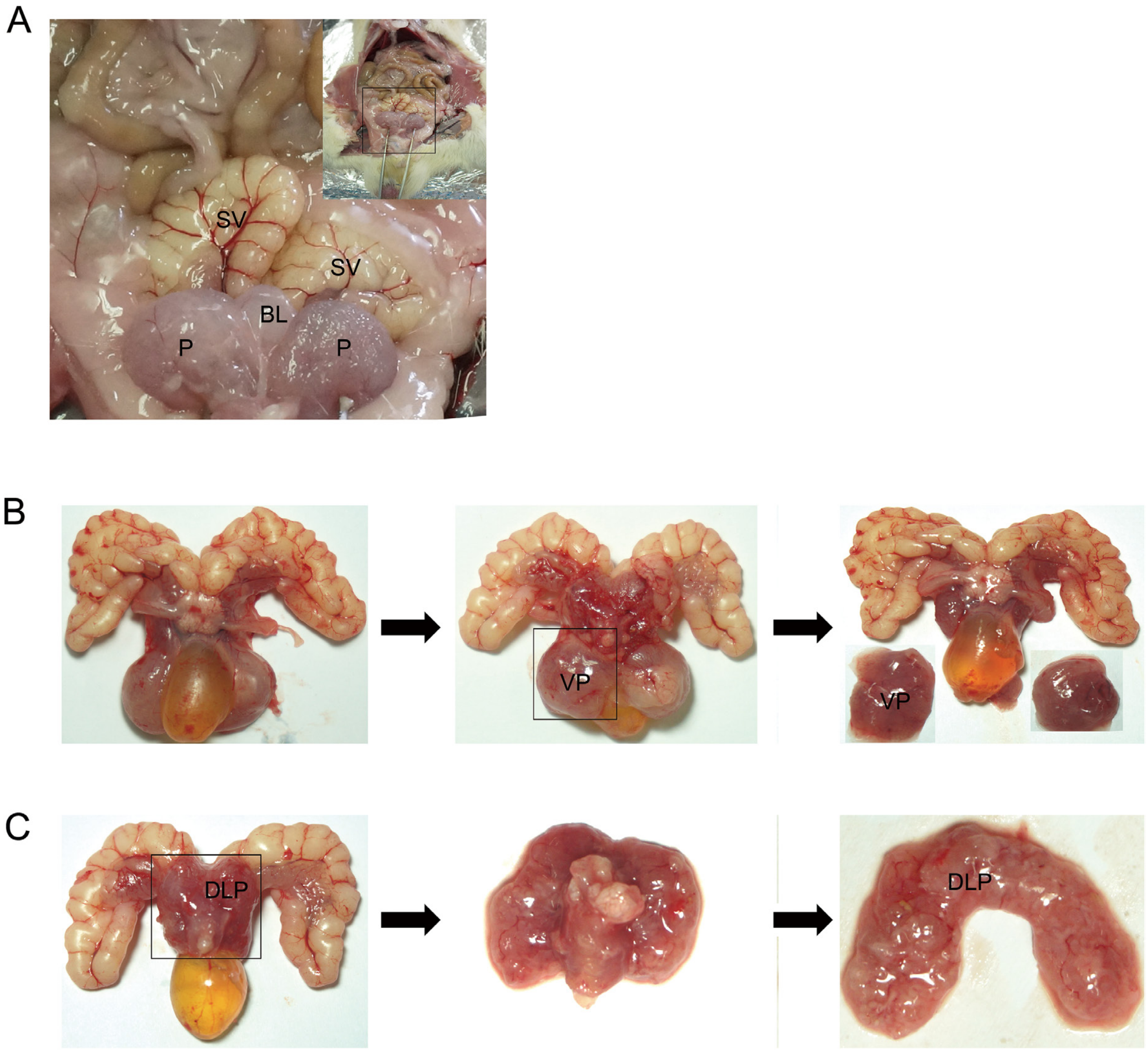

Supplementary Figure 1: The location and dissection of the prostate. **(A)** Genital organs in the abdominal cavity (SV, seminal vesicle; BL, bladder; P, prostate). **(B)** Ventral view of the isolated organs (VP, ventral prostate) and **(C)** dorsal view of the isolated organs (DLP, dorsolateral prostate). The dissection proceeded as stated in the steps.
